# Assessing Variability of Infiltration Characteristics and Reliability of Infiltration Models in a Tropical Sub-humid Region of India

**DOI:** 10.1038/s41598-020-58333-8

**Published:** 2020-01-30

**Authors:** Smaranika Mahapatra, Madan K. Jha, Sabinaya Biswal, Debasis Senapati

**Affiliations:** 1 AgFE Department, IIT Kharagpur, Kharagpur, 721 302 West Bengal India; 2 Department of Water Resource Development & Management, IIT Roorkee, Roorkee, Uttarakhand India

**Keywords:** Hydrology, Civil engineering

## Abstract

Infiltration process, which plays a paramount role in irrigation and drainage systems design, groundwater recharge and contamination evaluation, flood and drought management etc. is often controlled by several factors, among which land use/land cover (LULC) and soil physical properties are the prime factors. These factors lead to significant spatial variability of infiltration process, which poses a serious challenge for hydrologists and water managers. However, studies analyzing spatial variability and influence of both LULC and soil physical properties are scarce. To this end, grid-based infiltration experiments were carried out in a tropical sub-humid region of India to investigate spatial variability of infiltration characteristics, saturated hydraulic conductivity (K_sat_) as well as to evaluate reliability of seven infiltration models in predicting infiltration behaviour and estimating K_sat_. Additionally, uncertainty analysis was performed to quantify uncertainties associated with estimated K_sat_ for different LULC and soils. Results indicated that quasi-steady infiltration rate over the study area vary considerably with a majority of the area falling under ‘low’ and ‘medium’ infiltration categories. The infiltration process is greatly influenced by macro-pores and relatively low-permeable layers present at varying depths, typical features of lateritic vadose zones in tropical sub-humid regions, rather than its sole dependence on texture and LULC. Further, the Brutsaert model estimates K_sat_ with the highest accuracy and least uncertainty followed by Swartzendruber and Horton models. Except the Brutsaert model, other models are sensitive to a particular LULC. Overall, it is inferred that the Brutsaert and Swartzendruber models are robust and more reliable in predicting infiltration behavior and K_sat_ for the area. Findings of this study including quantification of spatial variability of important soil properties are useful for understanding detailed hydrological processes in the region and thereby, ensuring better planning and management of recurring floods and drought problems of the region.

## Introduction

Rapidly growing global population has led to increased demands for food, water and land. On top of it, changing climate severely affects the balance between demands and supply of water resources leaving limited source of water available for agriculture worldwide, especially in developing nations. To meet the present and future food demand, there is a need to increase the food-grain productivity with the limited availability of land and water resources. It has been estimated that irrigated agriculture accounts for 40% of global food production, whereas it accounts for more than 70% of the food grain production in India^1^. In fact, many studies^2,3^ have focused on increasing the crop yield per unit water use as even a small increase in water-use efficiency could save a considerable amount of water and thus, can augment food production. To achieve this goal, efficient design of on-farm irrigation systems is essential, where soil infiltration characteristics play a crucial role in ensuring high water-use efficiency, optimal fertilizer application and efficient irrigation scheduling^4,5^. Infiltration, an important component of the water cycle, refers to the downward movement of water from the ground surface to the soil profile. It plays a pivotal role in irrigation and drainage systems design, groundwater recharge assessment, subsurface flow and contaminant transport investigation and modeling as well as hydrologic-systems design and modeling.

Groundwater serves as a vital source of water for drinking and agriculture purposes. In India, over 65% of irrigation water and 85% of drinking water supplies come solely from groundwater resources^6^. However, despite of its immense importance, groundwater crisis prevails in India^7^, which calls for an urgent attention and dedicated actions. Sustainability of this precious resource depends mainly on the quantity and quality of infiltrated water (surface and atmospheric water) reaching aquifers (groundwater reserve). Vadose zone, a buffer zone between the land surface and the zone of saturation predominantly controls the quantity and quality of water reaching the groundwater system. Thus, in-depth knowledge of infiltration process and vadose-zone characteristics is inevitable for the sustainable management of water resources.

While designing an irrigation system at a larger scale, irrigation engineers/water managers face serious difficulties mostly due to the greater spatial variability of infiltration process. To overcome this problem, several geostatistical techniques, such as kriging, inverse distance weighting, spline, etc. have been developed that can estimate spatial variability of soil hydraulic parameters. However, they require a large number of measured points/sites for reliable results, thereby limiting their use under data-scarce conditions. Further, given the strong heterogeneity of soil/vadose-zone systems, the accurate field measurement of infiltration is a daunting task, labor intensive and costly in practice, particularly at a large scale. As a result, modeling of infiltration behavior has received a great deal of attention by several researchers across the world, leading to the development of numerous analytical models. A robust infiltration model is of utmost importance for estimating soil infiltration capacity with a high accuracy as well as for the effective planning and management of surface water and groundwater resources. A review of the infiltration models along with their advantages and limitations is presented in Philip^8^, Swartzendruber and Youngs^9^, and Ravi and Williams^10^.

In the past, several infiltration-based studies have been carried out worldwide. These studies have focused on the aspects such as spatial variability of soil infiltration behavior, laboratory-based experiments, field experiments with different experimental conditions, and performance evaluation of different infiltration models^11–22^.

Moreover, as mentioned earlier, the infiltration process is controlled by several factors, among which, land use/land cover^23^ and soil physical properties^24^ are often considered as the prime factors affecting infiltration behavior and its spatial variability. In the recent past, a number of studies have been carried out to analyze the effects of land use/land cover change and land management practices on soil infiltration characteristics^25–33^. For instance, Shukla *et al*.^25^ found a significant effect of land use change and soil-management treatments on soil structure and infiltration characteristics in the north Appalachian region of Ohio. In a recent study, Sun *et al*.^32^ analyzed the effect of land use change on the soil infiltration capacity using published infiltration data from different parts of China. Their results showed a reduced rate of infiltration after converting the land cover from grassland, shrubland and forest to cropland.

On the other hand, some researchers analyzed the effect of soil texture on the soil infiltration characteristics^34–36^. A few researchers studied the impacts of soil texture, structure, sodicity, bulk density and organic content on infiltration dynamics^37–39^. For example, Mirzaee *et al*.^35^ evaluated the impacts of soil textural classes and the ability of eight infiltration models to predict infiltration characteristics of four soil textural classes, viz., loam, silt loam, clay loam and silty clay loam. It was revealed that the soil textural classes have significant impacts on the performance of infiltration models and the Revised Kostiakov-Lewis model with four parameters fitted well with the cumulative infiltration data of loam, clay loam and silty clay loam soils, while the Kostiakov-Lewis model performed best for silty loam soils. However, very few studies are reported in the literature, wherein the effects of both soil physical properties and land use/land cover on soil infiltration characteristics have been investigated^40,41^. Shao and Baumgartl^40^ investigated the potential impacts of soil, vegetation, topography, and rainfall intensity on the performance of four infiltration models (Philip, Green–Ampt, Horton, and Holtan models) using the infiltration rate of 28 plots obtained by rainfall simulator. The results indicated that the performance of the Holtan and Horton models was better than that of physically based Green-Ampt and Philip models. In a recent study, de Almeida *et al*.^41^ evaluated the effects of tillage practices (conventional and no-tillage) and land use/land cover change on soil infiltrability and assessed the performance of infiltration models. It was found that the soil infiltration characteristics are more sensitive to the land use types than the soil tillage practices. Further, among the Kostiakov-Lewis, Horton and Philip models, the Horton model performed the best.

From the above literature survey, it can be inferred that limited studies have explored the effects of land use/land cover and soil texture on soil infiltration characteristics and the performance of infiltration models. Also, no studies deal with the impacts of land use/land cover and soil texture on the soil infiltration characteristics of lateritic vadose zones, typically found in tropical sub-humid/humid regions. It is worth to mention that the lateritic vadose zones are formed due to the laterization process in tropical humid/sub-humid regions that makes the soil soft and sticky when wet, and hard when dry, leading to the formation of impermeable/low-permeability layers at varying depths. Consequently, the flow through lateritic vadose zones is much more complex than other vadose zones. Laterization also causes prolonged perching condition along with dominant lateral flow during rainy seasons, which further complicates vadose-zone flow dynamics. Further, heterogeneity is an inherent characteristic of any porous media, due to which both physical and hydraulic properties of porous media vary appreciably from one location to another. Such wider scale variability over the land surface as well as below the ground (vadose zone) poses one of the greater challenges for irrigation and drainage engineers, soil scientists, hydrologists and hydrogeologists as well as for concerned planners and decision makers. Therefore, there is an urgent need to address this issue at a larger scale as well as to quantify spatial variability at a sub-basin or basin scale in order to solve real-world water problems.

Cognizant of the need for improved understanding and reliable information about the infiltration characteristics of lateritic vadose zones, typically found in tropical humid/sub-humid regions, the present study was conceived with three-fold objectives: (i) to quantify spatial variability of infiltration characteristics and hydraulic conductivity based on grid-scale field experiments in the study area, (ii) to evaluate the performance of salient infiltration models in predicting cumulative infiltration, and (iii) to assess the reliability of salient infiltration models for estimating saturated hydraulic conductivity along with an uncertainty analysis. To achieve these goals, infiltration tests were carried out at a grid scale in the Campus of Indian Institute of Technology Kharagpur (study area) under different land use/land cover and soil types. The study area experiences a tropical sub-humid climate and represents a typical lateritic belt of Eastern India. Outcomes of this study will provide an insight into the infiltration dynamics of lateritic vadose zones, which in turn will aid in improving water-use efficiency, sustainable groundwater management and efficient stormwater management in the region.

## Methodology

### Study area

**Figure 1:**
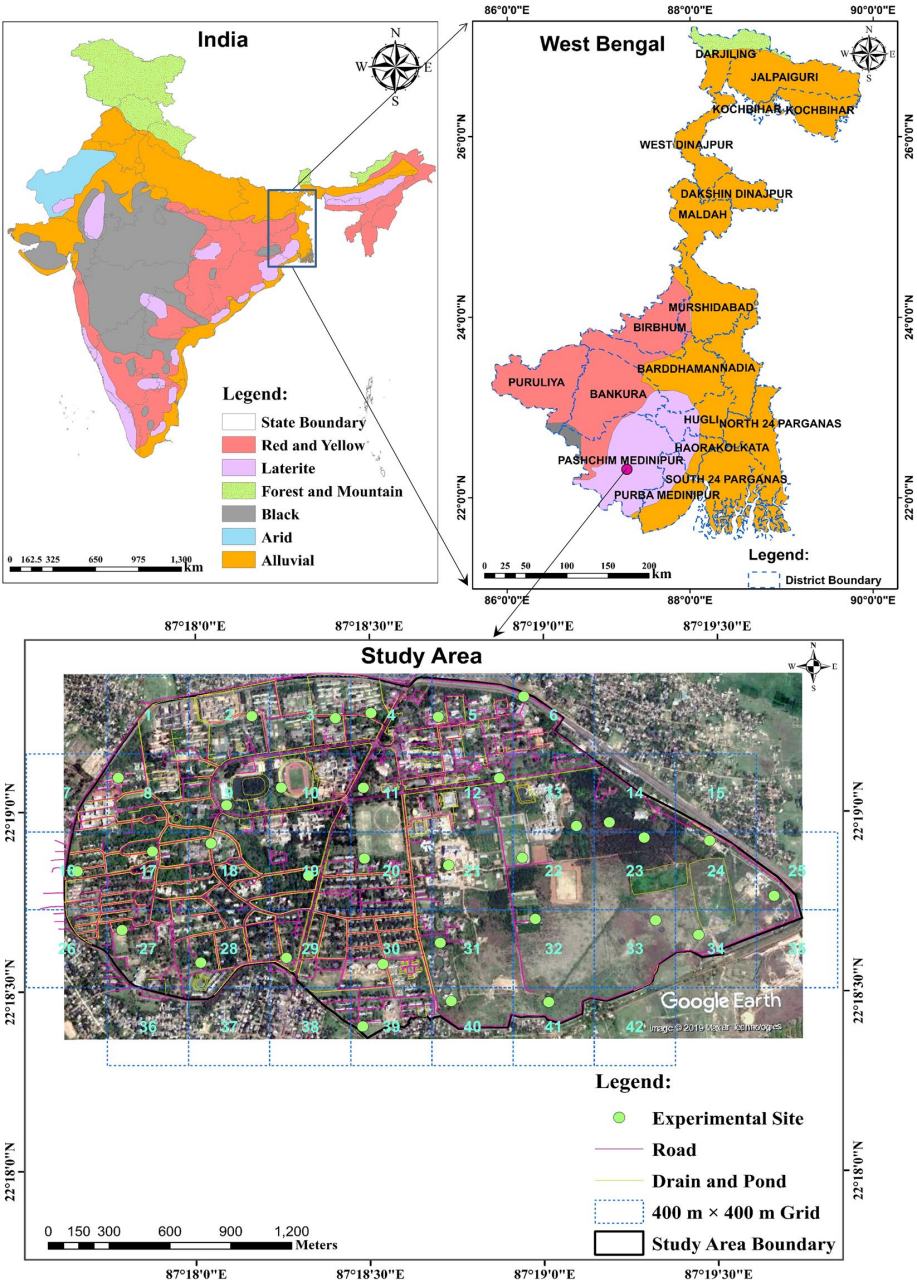
Map showing location of the study area and the experimental sites (indicated as green dots) within 33 square grids covering the study area. Remaining grids were excluded due to their location near the boundary (covering very less area) and/or unavailability of suitable sites for field experiments. Map of India was modified from the original image obtained from https://nroer.gov.in/5645d28d81fccb60f166681d/file/57cff5fe16b51c038dedcb21 [CC BY-SA 4.0 (https://creativecommons.org/licenses/by-sa/4.0/)]; country, state and district boundary shapefiles were downloaded from http://diva-gis.org/datadown. The satellite image of the study area was exported from Google Earth Pro version 7.3 (https://www.google.com/earth) and the final map was prepared in ArcMap version 10.1 (http://www.desktop.arcgis.com/en/arcmap/) along with roads, drainage networks and ponds.

The area selected for this study is the campus of Indian Institute of Technology (IIT) Kharagpur, which is located in the West Bengal state of Eastern India (Fig. 1). It lies between 22°19′10.97″N latitude and 87°18′35.87″E longitude and encompasses an area of 850 ha (8.5 km^2^). The study area falls in the lateritic region of Eastern India, and it has a moderately flat topography with the land elevation varying from 36 to 58 m MSL. Climate of the study area is characterized as tropical and sub-humid. It experiences hot and humid weather during summer seasons (temperature ranging from 37 to 46 °C), and cold and dry weather during winter seasons (temperature ranging from 8 to 10 °C). More than 80% of the average annual rainfall (about 1400 mm) occurs during June to October.

The study area usually suffers from surface flooding in monsoon seasons due to short-duration and high-intensity rainfalls. Additionally, groundwater being the main source of water supply for fulfilling the drinking and agriculture demands in the study area, its sustainability is threatened due to heavy exploitation of groundwater and gradual increase in the built-up area, thereby diminishing opportunities for natural recharge. To address these water problems of the study area, efficient drainage systems coupled with technically sound strategies for groundwater management are essential, for which infiltration plays a key role. This study, therefore, focuses on the detailed investigation of infiltration process in the study area.

### Field investigations

#### Determination of soil physical properties and hydraulic properties

For field investigations, the study area was divided into 42 square grids, each having a dimension of 400 m × 400 m (Fig. 1). However, experiments were conducted in 33 grids which cover a majority of the study area. The remaining grids (Grids: 1, 7, 15, 26, 35, 36, 37, 38, and 42) were excluded due to their location near the study area boundary (covering very less area) and/or unavailability of suitable sites for conducting experiments. Considering depth to water table below the ground surface at various locations over the study area, the thickness of vadose zone varies from about 5 to 8 m. Based on past investigations in the study area, maximum advancement of wetting front during infiltration experiments is generally limited to 0.30 m below the ground surface. Given this fact and the ease in collecting undisturbed core samples, soil samples were collected from the top vadose-zone layer (0–0.30 m) in each grid to investigate soil physical properties (i.e., soil texture and bulk density) and saturated hydraulic conductivity of the soil. Two sets of undisturbed soil samples were collected from each of the 33 grids of the study area (resulting in 66 soil samples) to determine bulk density and saturated hydraulic conductivity in the laboratory. Afterwards, the percentage of sand, silt and clay was determined by the pipette method in the laboratory. The texture of all the soil samples were identified based on the USDA Soil Texture Classification System^42^. Maps showing spatial variation of soil texture and bulk density over the study area were also generated using ArcGIS 10.1.

Moreover, using the second set of undisturbed soil samples, saturated hydraulic conductivity was determined by constant head permeameter method in the laboratory. With careful inspections for loosening of soils during core sampling, only the intact soil samples were fully saturated in the laboratory. Thereafter, the saturated soil samples were imposed to a hydraulic-head difference and the resulting flux of water was measured until a steady flow rate was obtained. The saturated hydraulic conductivity (henceforth called ‘measured K_s_’) of the soil samples was measured under a constant head. The time required to achieve a steady-state flow condition through the soil columns varied from about 1 to 5 h.

#### Infiltration experimentation

In the present study, infiltration tests were conducted during the months of November and December, 2016 in all the 33 grids using double ring infiltrometers to quantify the spatial variability of infiltration process and study the infiltration dynamics. Location of the infiltration-test sites within the grids are shown in Fig. 1. Double ring infiltrometers having inner ring and outer ring diameters of 30 and 60 cm, respectively were inserted carefully to approximately 10 cm depth at each grid. Utmost care was taken to minimize the disturbance of the soil surface inside the rings at the time of pouring water into the rings. Initially, the rate of fall of water level in the inner ring was measured after 5 minutes and for the next half an hour, the readings were taken at every 5-minute time interval. Afterwards, the time interval was increased to 10 minutes for the next one hour, followed by 15 minute-interval up to the period of two hours, 30-minute interval up to two hours, and finally 60-minute interval up to end of the test. However, these measurement intervals were kept flexible according to site-specific infiltration rates. All the tests were terminated after attaining a steady/quasi-steady infiltration of water at each location. Further, quasi-steady infiltration rates (i.e., basic infiltration rates) for individual grids were determined from the infiltration rate versus time plots for all the grids. The quasi-steady infiltration rates obtained represent field-saturated hydraulic conductivity values (*K*_*fs*_), which are considered as the observed field saturated hydraulic conductivity in this study.

#### Preparation of land use/land cover map

Land use/land cover map of the study area was prepared from LISS-IV (15th January 2016) (Resourcesat-2, NRSC, Hyderabad, India) satellite image using supervised classification system in the ArcGIS 10.1 environment. The land use/land cover map thus obtained was verified using the Google Earth imagery and detailed ground truth data. The wrongly classified pixels in the map were further corrected according to the ground truth data collected and a final land use/land cover map was prepared to explore the major land use and land cover types in the study area.

### Fitting of infiltration models to field data

#### Infiltration models

In this study, seven infiltration models well-established in the literature – two empirical models (viz., Kostiakov-Lewis and Horton) and five process-based models (viz., Philip Two-Term, Kutílek and Krejča, Swartzendruber, Stroosnijder, and Brutsaert) were selected to evaluate their efficacy in replicating the infiltration behavior of the study area. The infiltration models used in this study are briefly discussed below.

Kostiakov-Lewis model. The Kostiakov infiltration model has a limitation in predicting infiltration behavior at a larger time step. To overcome this limitation, the Kostiakov equation was modified by adding a constant, the ‘steady infiltration rate’ term. This modified form of the Kostiakov model is popularly known as Kostiakov-Lewis model or Mezencev’s model. The Kostiakov-Lewis model for ‘cumulative infiltration’ is given as:

1 $$I(t)=\frac{a}{1-b}{t}^{1-b}+{i}_{b}t$$

where *I* = cumulative infiltration at time *t, a* and *b* are empirical parameters and *i*_*b*_ = ‘quasi-steady infiltration rate’ representing saturated hydraulic conductivity.

Horton model. Horton model is one of the most popularly used empirical infiltration equations. Horton^43^ presented a three-parameter empirical infiltration model, popularly used in hydrologic modeling. He observed that the infiltration rate at the beginning of a storm event decreases exponentially with time until it reaches a more or less constant rate^44,45^. The Horton equation for cumulative infiltration is as follows:

2 $$I(t)=\frac{{i}_{0}-{i}_{c}}{m}(1-{e}^{-mt})+{i}_{c}t$$

where *i*_0_ = initial infiltration rate; *i*_*c*_ = quasi-steady infiltration rate, providing an estimate of saturated hydraulic conductivity, and *m* = decay constant. In this study, the three parameters, *i*_*0*_, *i*_*c*_ and *m* were treated as the fitting parameters.

Application of the empirical models are limited by a major disadvantage that they do not provide insights into physical processes like process-based infiltration models.

Philip Two-Term model. Philip model^8^ was developed by solving the one-dimensional Richards equation. The truncated form of this infinite time series solution with its first two terms is known as ‘Philip Two-Term’ model. The mathematical expression of the Philip Two-Term model for ‘cumulative infiltration’ is given by:

3 $$I(t)={S}_{P}{t}^{0.5}+At$$

where *S*_*p*_ = soil sorptivity depending on initial soil water content and soil water diffusivity; and *A* = a parameter known as transmissivity factor.

Philip Two-Term model is limited by the fact that use of the first two terms of the infinite series solution leads to an inherent large truncation error in the parameter *A* of this model.

Kutílek and Krejča model. In order to minimize the truncation error, first three terms of the infinite time series solution of Philip^8^ were used by Kutílek and Krejča. The modified infiltration equation is known as ‘Kutílek and Krejča model’^46^ for ‘cumulative infiltration’ and is expressed as:

4 $$I(t)={C}_{1}{t}^{0.5}+{C}_{2}t+{C}_{3}{t}^{1.5}$$

where *C*_1_ = an estimate of soil sorptivity; *C*_2_ = an estimate of [(*A*_2_ + *K(θ*_*i*_)], and *C*_3_ = an estimate of (*A*_3_ + Truncation error). The truncation error involved in this model is lower than the Philip Two-Term model, thereby improving its fitting ability^17^. Using this model, the saturated hydraulic conductivity (*K*_*sk*_) can be computed as:

5 $${K}_{sk}=3\times {({C}_{1}\times {C}_{3})}^{0.5}+{C}_{2}$$

Despite of having less truncation error as compared to Philip Two-term model, application of the Kutílek and Krejča model is limited by the fact that it is more sensitive to heterogeneity in real field conditions. This model may yield unrealistic negative estimates of the parameters (C_1_, C_2_ and C_3_), resulting in unreliable values of saturated hydraulic conductivity^46,47^.

Swartzendruber model. Swartzendruber^48^ proposed an alternative series solution by adjusting the Philip’s infinite series solution. The modified infiltration time series is valid for all infiltration times^49^. This model also allows for surface ponding. This new infiltration time-series solution can be approximated as a three-parameter equation, which for ‘cumulative infiltration’ is expressed as follows:

6 $$I(t)=\frac{{S}_{SW}}{{A}_{0}}\times (1-{e}^{-{A}_{0}{t}^{0.5}})+{K}_{SW}t$$

where *S*_*sw*_ = soil sorptivity; *A*_0_ = parameter depending on surface water content; and *K*_*sw*_ = an estimate of saturated hydraulic conductivity.

Stroosnijder model. Substituting 4*K*_*str*_/3*S*_*str*_ for *A*_0_ in Eq. (6) yields a two-parameter Stroosnijder infiltration equation^50^, which is known as Stroosnijder model and it is expressed as:

7 $$I(t)=\frac{3{S}_{str}^{\,2}}{4{K}_{str}}\times \left[1-\exp \left(-\frac{4{K}_{str}{t}^{0.5}}{3{S}_{str}}\right)\right]+{K}_{str}t$$

where *S*_*str*_ = an estimate of soil sorptivity; and *K*_*str*_ = an estimate of saturated hydraulic conductivity.

Equation (7) approaches Philip solution behavior at moderate times and at very large times (t→ ∞), dI/dt approaches a finite steady-state infiltration rate^15,26^.

Brutsaert model. Brutsaert^51^ introduced a correction factor for the gravitational force and adjusted the Philip solution^46^. He derived the following three-parameter equation for ‘cumulative infiltration (I)’, which is known as Brutsaert model:

8 $$I(t)={K}_{b}t+\frac{{S}_{b}^{\,2}}{B{K}_{b}}\left[1-\frac{1}{1+\frac{BK{t}^{0.5}}{{S}_{b}}}\right]$$

where *K*_*b*_ = saturated hydraulic conductivity; *S*_*b*_ = soil sorptivity; and *B* = a dimensionless parameter depending on soil properties.

Inverse modeling. Cumulative infiltration time-series data obtained from the field experiments at each location/grid were fitted to the seven infiltration models [Eqs. (1–8)]. As recommended by Jha *et al*.^22^, only cumulative infiltration time series was considered in this study for infiltration model fitting. The infiltration-model parameters were optimized for each location by the ‘Trust Region’ algorithm, with the help of MATLAB (R2014a version) software. The model parameters representing saturated hydraulic conductivity and sorptivity (i.e., *i*_*b*_*, A, K*_*sk*_*, K*_*sw*_*, K*_*str*_, *K*_*b*_, *S*_*p*_*, C*_1_*, C*_2_*, C*_3_*, S*_*sw*_, *S*_*str*_, and *S*_*b*_) were searched in the domain (upper and lower bounds) of positive real numbers for obtaining realistic values. However, the remaining model parameters were optimized without restricting their lower and upper bounds during optimization. The objective function used to optimize the infiltration-model parameters is given as:

9 $${\rm{Minimize}},SSE=\mathop{\sum }\limits_{i=1}^{n}{[{({I}_{o})}_{i}-{({I}_{pred})}_{i}]}^{2}$$

where (*I*_*o*_)_*i*_ = observed cumulative infiltration obtained at the *i*^th^ time and (*I*_*pred*_)_*i*_ = predicted cumulative infiltration at the *i*^th^ time, and n = total number of measured data points.

Finally, the time series of cumulative infiltration were predicted by each infiltration model using the optimal parameters of the corresponding infiltration model.

### Evaluation of the infiltration models for predicting cumulative infiltration

The efficacy of the seven infiltration models in simulating cumulative infiltration was rigorously evaluated using both dimensional statistical indicator, viz., Root Mean Squared Error (RMSE) and non-dimensional statistical indicators such as Correlation Coefficient (r) and Nash-Sutcliffe Efficiency (NSE). Smaller values of RMSE, and higher values of r and NSE indicate a better agreement between measured and simulated values of ‘cumulative infiltration’. Generally, model performance improves with increase in the number of model parameters. However, other goodness-of-fit criteria (e.g., R^2^, NSE) do not consider the number of unknown parameters involved in the models. On the other hand, Akaike Information Criterion (AIC) takes the number of parameters to be estimated into account while evaluating the models for predicting cumulative infiltration. Some researchers have also used this criterion for comparing the models for predicting particle size distribution^52^, soil-hydraulic properties^53^, cumulative infiltration^20,35^. Therefore, AIC was used as an additional goodness-of-fit criterion in this study for un-biased assessment of the infiltration models. The original mathematical expression of AIC^54^ is given as:

10 $$AIC=(-2)\times \,\mathrm{ln}(L)+2k$$

where *L* = maximized value of the likelihood function; *k* = total number of estimated parameters of a model.

Substituting the value of *L* in Eq. (10), AIC was estimated from the sum of square error (SSE) as follows^53^:

11 $$AIC=N[\mathrm{ln}(2\pi )+\,\mathrm{ln}\{(SSE/(N-p)\}+1]+p$$

where *N* = number of observation points; and *p* = number of model parameters to be estimated.

Best infiltration model for predicting cumulative infiltration was selected based on the smallest value of AIC. Finally, grid-wise ranking of the models were performed based on individual statistical indicators. Overall ranking of a given infiltration model for the study area was decided based on its performance at majority of the grids (i.e., number of grids). Also, graphical indicator such as simultaneous plots of observed and predicted ‘cumulative infiltration’ was considered.

### Analysis of measured data and model parameters

This study has focused on only one parameter estimated by the infiltration models, i.e., saturated hydraulic conductivity. Kolmogorov-Smirnov test was applied for determining the statistical distribution of the ‘measured *K*_*s*_’, field-saturated hydraulic conductivity (*K*_*fs*_) and model-estimated saturated hydraulic conductivity values. The best-fit probability distribution for each parameter was chosen using Kolmogorov statistic. Furthermore, these parameters were analyzed statistically to investigate their spatial variability using one-way ANOVA (Analysis of Variance). A post-hoc analysis, Tukey’s honest significant difference (Tukey HSD) test was also carried out for pair-wise comparison of the model-estimated saturated hydraulic conductivity and the measured values as well as to assess the impacts of soil texture and land use/land cover on these parameters. The null hypothesis of the statistical tests was that the true difference of the means is equal to zero. All these statistical analyses were carried out in R software^55^ environment. Reliability of the infiltration models were also assessed based on their ability to estimate the saturated hydraulic conductivity by visual comparison with the corresponding measured values. Statistical indicators, viz., mean absolute error (MAE), RMSE and NSE were also calculated for evaluating their reliability in estimating the saturated hydraulic conductivity.

### Uncertainty analysis of infiltration models

A statistical analysis was performed with a goal of determining the uncertainty related with the seven infiltration models in estimating saturated hydraulic conductivity. For this purpose, non-parametric bootstrap method, a popular uncertainty analysis method was carried out. This method is particularly suitable for small-sample data sets where it is hard to find the statistical distribution of the sample. In the present study, confidence intervals were estimated around the mean of the saturated hydraulic conductivity estimated by a given infiltration model. Let the *x*_*i*_ be the original data series having *n* number of data points; *x*_*i*_ = *x*_1_*, x*_2_, *x*_3_*,…, x*_*n*_. Random sampling was done with replacement and without assuming any distribution of the parameters while implementing bootstrap method. Let *x*_i_^*^ be the random sample (size: *n*) generated from the original sample. The observations were assumed to be independent and sampling was repeated for 1000 times, as recommended for 95% confidence interval by Carpenter and Bithell^56^. Mean of each bootstrap sample (i.e., µ_i_^*^) was estimated. Finally, 1000 independent sets of bootstrap statistics (µ_i_^*^) were generated. Thereafter, lower and upper limits of the 95% confidence interval were derived by calculating the 2.5 and 97.5 percentile of the generated 1000 sets of µ_i_^*^ values. The procedure was repeated for each infiltration model for quantifying the uncertainty related with them in estimating saturated hydraulic conductivity for the entire study area. Furthermore, uncertainty analysis of the models was also carried out separately for varying soil textures and land use/land covers.

## Results and Discussion

### Soil physical properties and saturated hydraulic conductivity

**Figure 2:**
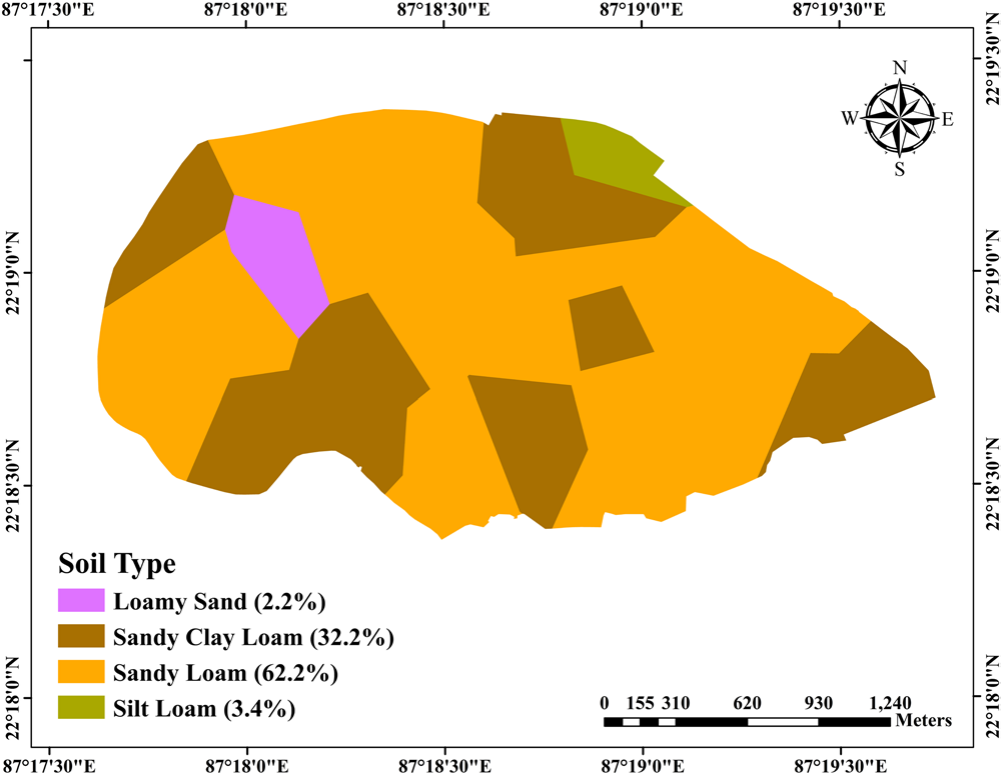
Soil map showing four major types of soil (top 0.30 m vadose zone) covering the study area and their percentage of coverage (values within parentheses).

**Figure 3:**
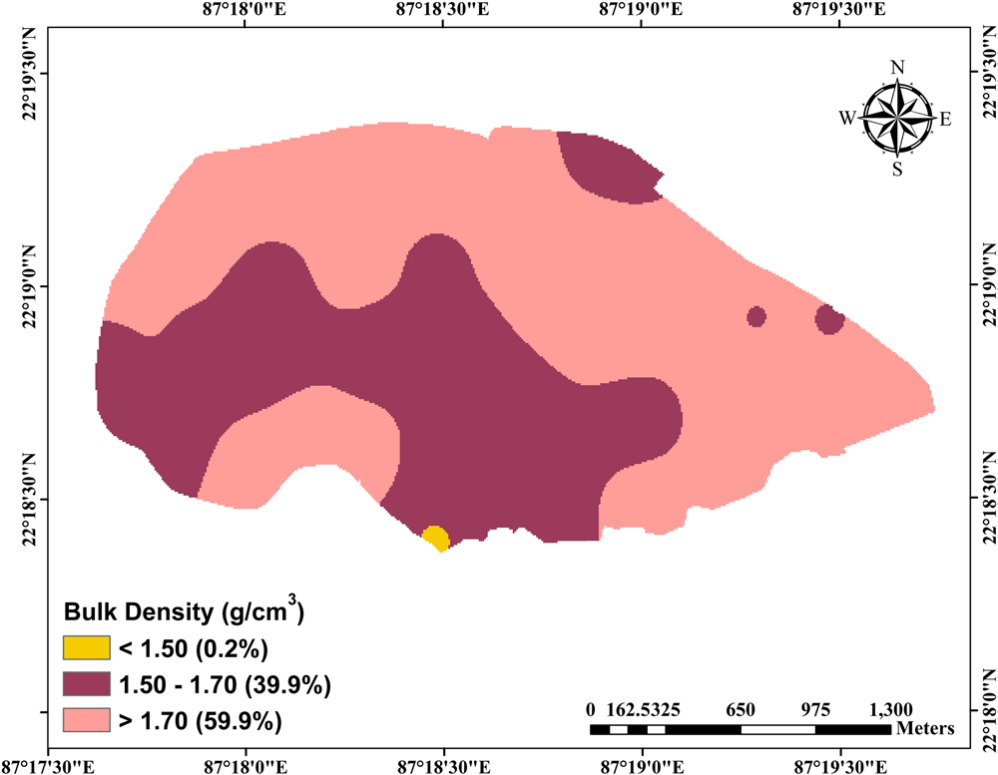
Spatial variation of bulk density (top 0.30 m vadose zone) over the study area. Values in parentheses indicate percentage area covered under each class.

The soil samples collected were analyzed for determining sand, silt, clay percentage, and bulk density of the top vadose-zone layer (0–0.3 m). The statistical description of the results obtained is summarized in Table 1. Additionally, the spatial variations of texture and bulk density are illustrated in Figs. 2 and 3, respectively. According to the USDA soil textural classification, top vadose-zone layer in the study area consists of four major soil types, viz., sandy loam (62.2% of the area), sandy clay loam (32.2%), silt loam (3.4%), and loamy sand (2.2%). However, they display discernible spatial variation in terms of sand, silt and clay contents (Table 1). The mean sand content in the study area is 69.7% (standard deviation: 11.1%) with a lesser coefficient of variation (CV) of 16%. In contrast, silt and clay content are found to be highly variable having CV values above 30%. Bulk density of the study area ranges from 1.47 to 1.94 g/cm^3^ having the least spatial variability (CV: 7.3%). Majority of the area (i.e., 59.9% of total area) have bulk density greater than 1.70 g/cm^3^, whereas 39.9% of the area has moderate bulk density values ranging from 1.5 to 1.70 g/cm^3^ and only 0.2% of the area has bulk density < 1.50 g/cm^3^ (Fig. 3). Furthermore, saturated hydraulic conductivity (‘measured *K*_*s*_’) of the study area varies significantly from 0.12 to 185.76 mm/h with an average value of 13.39** ± **34.28 mm/h (Table 1). It is also observed from Table 1 that among all the measured parameters, ‘measured *K*_*s*_’ shows the highest spatial variation with a CV value of 256%.

**Table 1:** General statistics of soil physical characteristics and saturated hydraulic conductivity of the study area.

SI. No.	Parameters	Statistics		
		Range	Mean ± SD	CV (%)
1	Sand (%)	26.0–82.0	69.7** ± **11.1	15.9
2	Silt (%)	4.0–64.0	13.0** ± **11.1	85.4
3	Clay (%)	6.0–28.0	17.4** ± **5.7	33.1
4	Bulk Density (g.cm^−3^)	1.47–1.94	1.72** ± **0.13	7.54
5	Measured *K*_*s*_ (mm/h)	0.12–185.76	13.39** ± **34.28	256.0

**Note:** SD: standard deviation; CV: coefficient of variation.

### Land use/land cover of the study area

**Figure 4:**
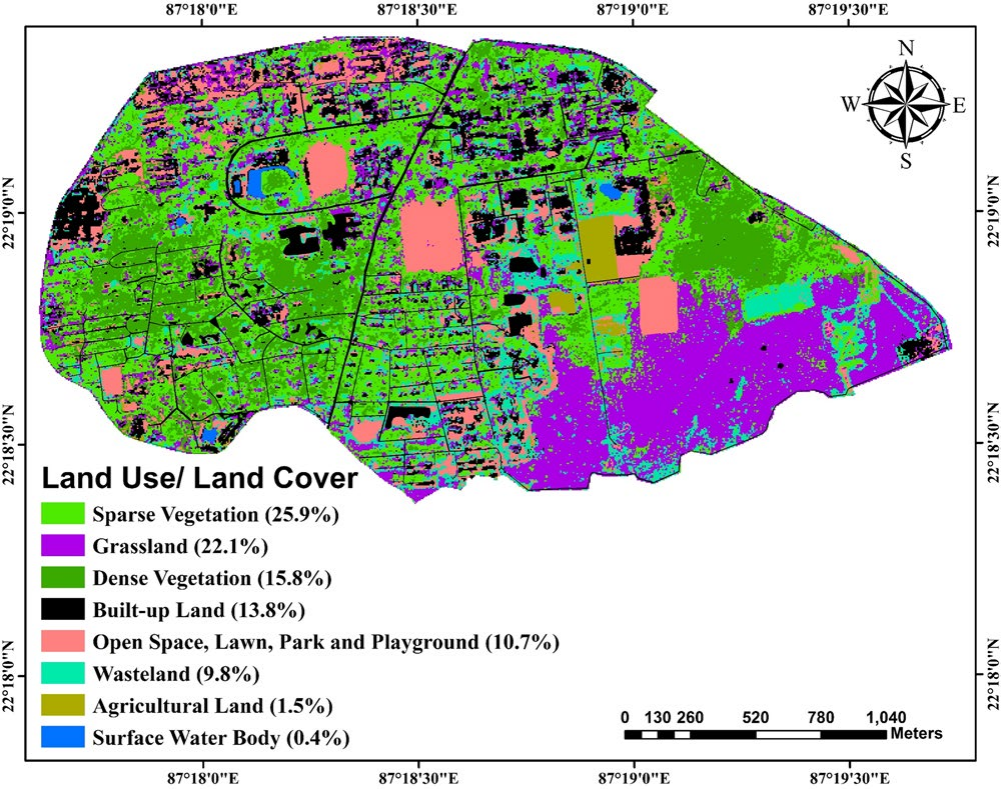
Land use/land cover map of the study area showing eight major classes. The percentage area covered under each class is given in parentheses.

Land use/land cover of the study area can be classified into eight classes, namely, sparse vegetation, grassland, dense vegetation, settlements, open space (including park, lawn and playground), wasteland, agricultural land, surface water body (Fig. 4). The details about the percentage of coverage of these major land use/land cover classes are also illustrated in Fig. 4. It is apparent that majority of the area is dominated by sparse and dense vegetation (about 42%) followed by grassland, which covers about 22% of the total area. Built-up land and open spaces (including park, lawn and playground) cover about 66 ha (about 14%) and 51 ha (about 11%), respectively. Area covered with agricultural activities is very less covering only 1.5% of the total area.

### Spatial variation of infiltration characteristics

**Figure 5:**
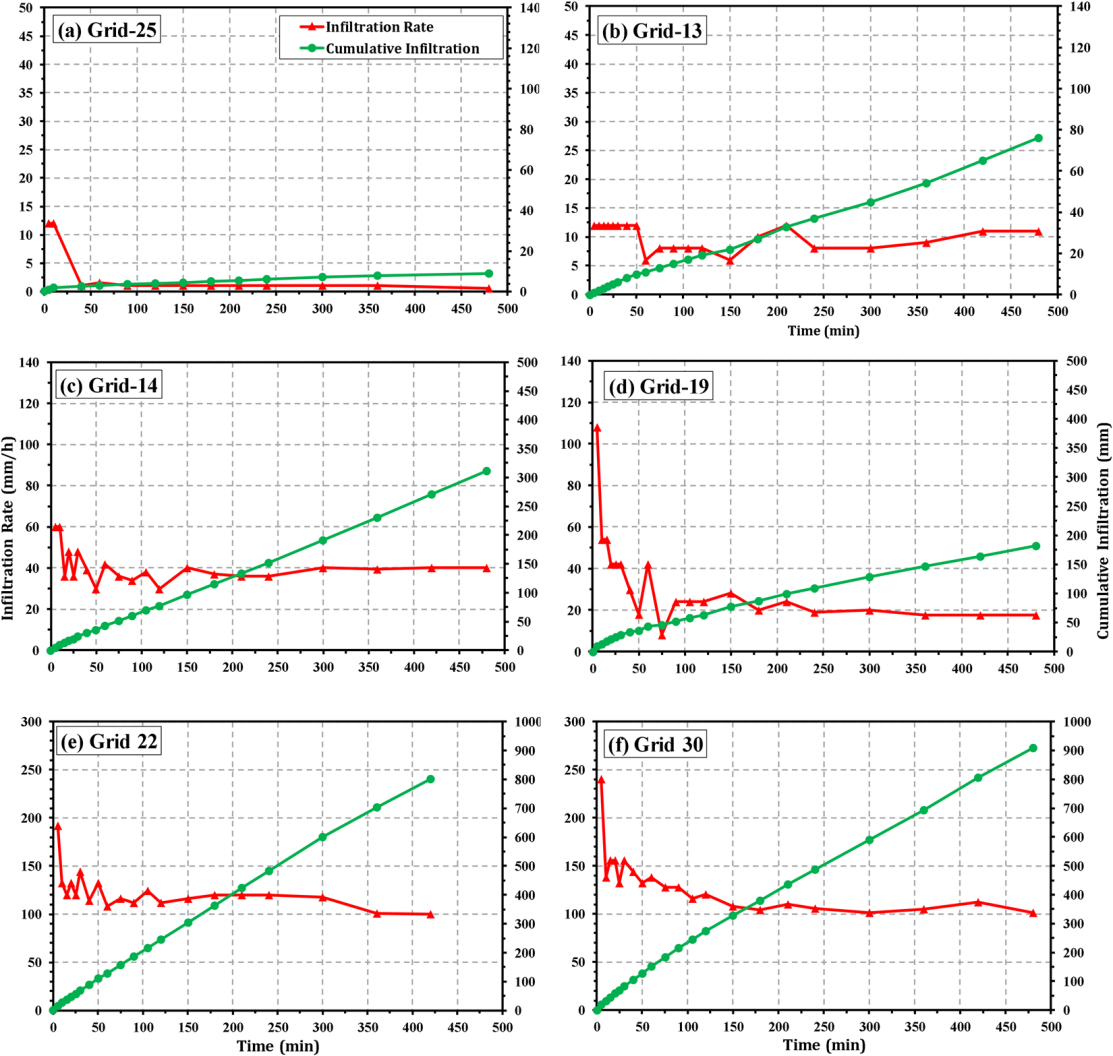
Infiltration characteristic curves at six grids of the study area representing *‘low’* basic infiltration rate [(**a**) Grid 25, (**b**) Grid 13], *‘medium’* basic infiltration rate [(**c**) Grid 14, (**d**) Grid 19], and *‘high’* basic infiltration rate [(**e**) Grid 22, and (**f**) Grid 30] classes.

The infiltration characteristics of the study area are illustrated in Fig. 5(a–f) for six grids/locations as an example. It is evident from the figures that sudden rise and fall of infiltration rate occurs at all locations. Such abrupt fluctuations in the infiltration rate suggest existence of macropores in the soil profile or sudden release of the entrapped air in the soil aggregate. Besides, a sudden decrease in the infiltration rate observed during the infiltration measurements may be caused by significant perching phenomena, often predominant in the lateritic vadose zone. This observation corroborates with the findings of Machiwal *et al*.^14^ and Jha *et al*.^22^ in a wasteland and cultivated lands of IIT Kharagpur, respectively.

**Figure 6:**
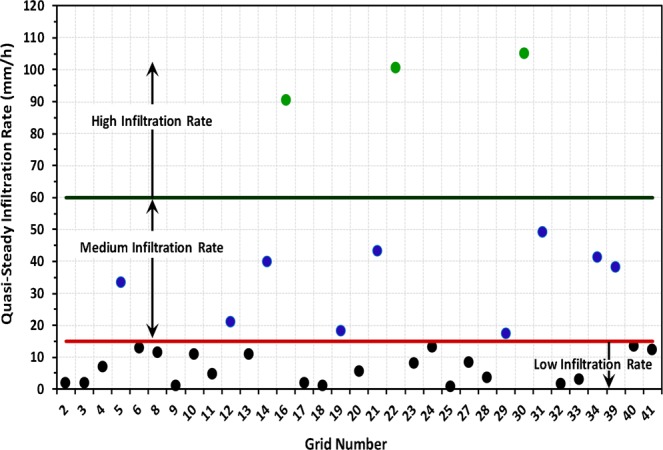
Grid-wise quasi-steady infiltration rate over the study area. Black solid circles indicate *‘low’* infiltration rate, blue solid circles indicate *‘medium’* infiltration rate, and green solid circles indicate *‘high’* infiltration rate.

**Figure 7:**
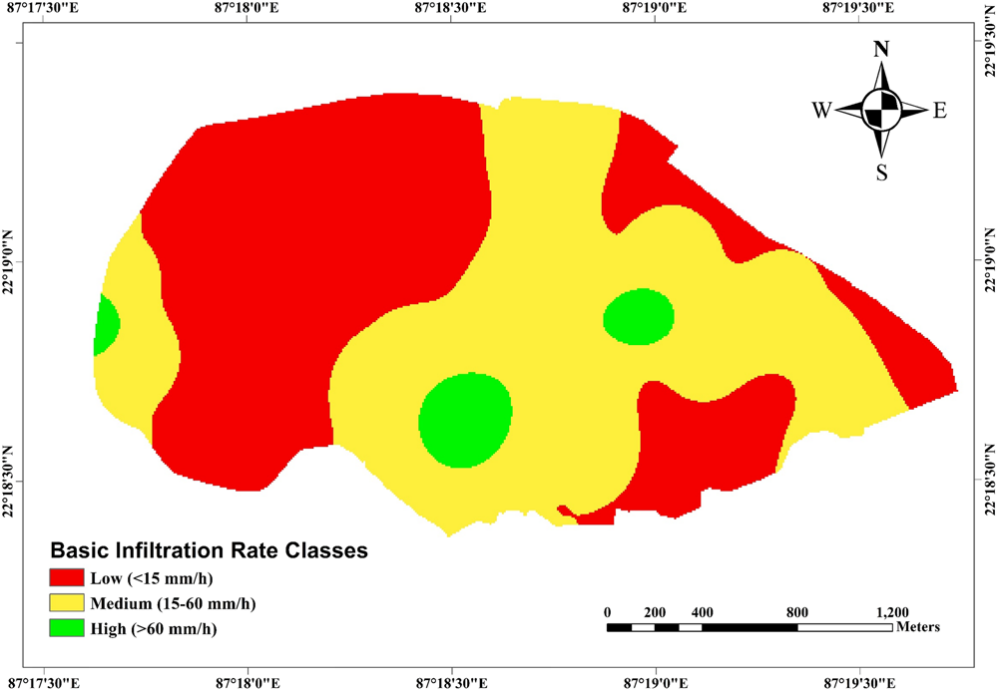
Spatial variation of “basic (quasi-steady) infiltration rate” over the study area.

Moreover, ‘quasi-steady infiltration rate’ i.e., ‘basic infiltration rate’ values of the 33 grids over the study area are shown in Fig. 6. It is obvious from Fig. 6 that quasi-steady infiltration rates (i.e., *K*_*fs*_) of the study area vary substantially with values ranging over two orders of magnitude, from 0.83 mm/h (Grid: 25) to 105.13 mm/h (Grid: 30), with a mean value of 22.23 ± 28.27 mm/h. As a result, total water infiltrated (i.e., cumulative infiltration at the end of the test) into the soil ranges from 9 to 908 mm during the infiltration experiments. The lowest value of the quasi-steady infiltration rate and cumulative infiltration at Grid: 25 could be attributed to the presence of very hard *murrum* content just 5 cm below the topsoil of the particular location/grid. The coefficient of variation (CV) of the infiltration measurements is very high (~127%) manifesting the heterogeneity and complexity of hydraulic processes in the lateritic vadose zone of the study area. Further, the values of quasi-steady infiltration rate obtained from the infiltration tests are classified into three categories, viz., *‘low’* (<15 mm/h), *‘medium’* (15–60 mm/h) and *‘high’* (>60 mm/h) following the standard guidelines of Food and Agriculture Organization (FAO). It is evident from Fig. 7 that a majority of the study area comes under *‘low’* infiltration rate and *‘medium’* infiltration rate classes, covering an area of 222.35 ha (i.e., 47.7% of the study area) and 223.79 ha (i.e., 48% of the area), respectively. On the other hand, the zones having *‘high’* infiltration rate are in scattered patches over the study area encompassing about 4% of the total area.

Furthermore, a comparison of the quasi-steady infiltration rate, which is equivalent to the field-saturated hydraulic conductivity (*K*_*fs*_), with the ‘measured *K*_*s*_’ of the study area revealed that *K*_*fs*_ values differ from ‘measured *K*_*s*_’ by several folds in all grids. At about 80% of the grids, larger values of *K*_*fs*_ are observed about 1 (in grids 3, 4, 12 and 13) to 300 times (at grid 22) of the ‘measured *K*_*s*_’, while about one (at grids 1 and 18) to three fold (at grids 28, 32, and 41) smaller values of *K*_*fs*_ are observed in the remaining grids. However, the difference between the mean values of *K*_*fs*_ and ‘measured *K*_*s*_’ is not statistically significant at 5% level of significance.

### Effect of soil texture and land use/land cover on infiltration characteristics

Effects of soil texture (two major classes, viz., sandy loam and sandy clay loam) and land use/land cover (four major classes, viz., orchard, grassland, shrubland, and vegetation) on the quasi-steady infiltration rate (*K*_*fs*_) are not found to be statistically significant at 5% level of significance as revealed from ANOVA tests. However, from a practical point of view, there is a considerable variation in the values of quasi-steady infiltration rate of the vadose zone having different soil textures and land use/land cover classes. For example, mean *K*_*fs*_ for sandy loam soil is 20.19 ± 23.95 mm/h, while mean *K*_*fs*_ for sandy clay loam soil is 30.70 ± 30.95 mm/h. Hard-murrum contents were visible beneath top 5 cm soil at some locations during the field investigations, which might have contributed to the low value of mean *K*_*fs*_ in the sandy loam soils. In contrast, several other researchers have reported very high values of quasi-steady infiltration rates for sandy loam type soils in different regions across the globe. For example, Alagna *et al*.^57^ found very high values of saturated hydraulic conductivity (>169 mm/h) derived from infiltration tests for sandy loam soils in Italy. For orchard and grassland, mean values of *K*_*fs*_ are 22.32 ± 20.85 mm/h and 23.57 ± 29.72 mm/h, respectively. On the other hand, lesser values of *K*_*fs*_ are found for shrubland and vegetation type lands having mean values of *K*_*fs*_ as 11.75 ± 19.63 mm/h and 12.30 ± 16.46 mm/h, respectively. The correlation analysis revealed that the quasi-steady infiltration rate (*K*_*fs*_) is weakly related to the texture and bulk density. This may be attributed to the variation in infiltration dynamics due to factors like presence of macropores and/or complex flow dynamics in lateritic vadose zones, rather than the direct dependency of infiltration behavior on texture, bulk density and land use/land cover only. Some researchers have also reported similar findings about the weak relationship between saturated hydraulic conductivity and soil textural compositions^58,59^. However, Rahmati *et al*.^60^ have reported a strong connection between saturated hydraulic conductivity and particle-size distribution. Nevertheless, a detailed investigation focusing on the influence of the above factors on the infiltration behavior of lateritic terrains is needed in the future.

### Performance of the infiltration models for predicting cumulative infiltration

**Figure 8:**
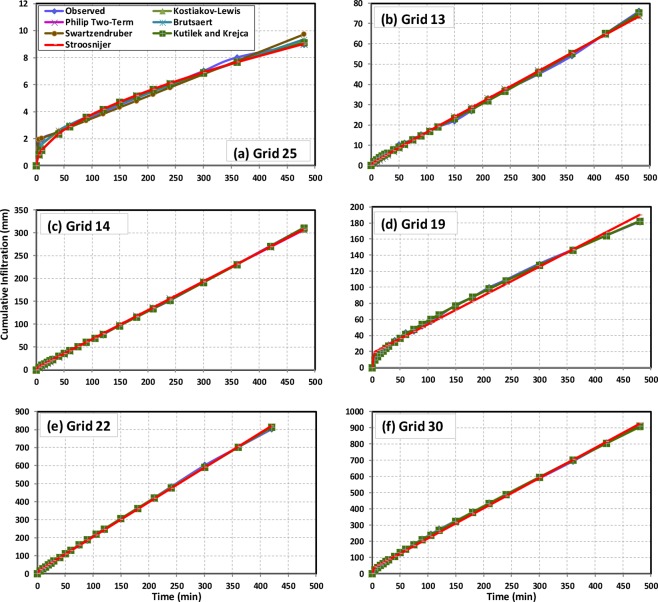
Plots of observed vs. predicted cumulative infiltration at six grids of the study area representing *‘low’* basic infiltration rate [(**a**) Grid 25, (**b**) Grid 13], *‘medium’* basic infiltration rate [(**c**) Grid 14, (**d**) Grid 19], and *‘high’* basic infiltration rate [(**e**) Grid 22, and (**f**) Grid 30] classes.

For most of the grids, all the infiltration models perform well in predicting cumulative infiltration. An illustration of the predictability of all the seven infiltration models for ‘cumulative infiltration’ at six grids is given in Fig. 8(a–f), as an example. It is discernible from the figures that all the infiltration models predict cumulative infiltration with high accuracy at most of the sites. However, the Stroosnijder model performs relatively poor as compared to the rest of the models with slight under-predictions and over-predictions at some grids; e.g., Grid 25 and Grid 19 (Fig. 8(a,d)).

Moreover, the mean values of r, RMSE and NSE at 33 grids of the seven infiltration models along with their ranges are summarized in Table 2. It is revealed from the table that all the models perform efficiently with very less errors (RMSE) ranging from 0.1473 to 45.9205 mm, while very high values of r (0.9622–1.0000) and moderate to very good values of model efficiency (NSE: 0.7569–1.0000) indicating that these infiltration models are well capable of predicting cumulative infiltration at all sites. Regarding the RMSE criteria, the Kostiakov-Lewis model has the least mean RMSE (1.3710 mm) in simulating cumulative infiltration at all the sites ranging from 0.1863 to 5.4202, followed by the Brutsaert (mean RMSE = 1.4934 mm; range = 7.1334 mm) and the Swartzendruber (mean RMSE = 1.5892 mm; range = 7.9727 mm) models. In contrast, the Stroosnijder model predicts cumulative infiltration with the highest mean error of 4.1198 mm and wider range in RMSE as 16.4213 mm (Table 2). Based on the RMSE values, the Philip Two-Term and the Kutílek and Krejča models perform more or less equally in predicting cumulative infiltration with the mean RMSE values of 2.1918 and 2.0352 mm, respectively.

**Table 2:** Goodness-of-fit statistics for predicting cumulative infiltration by the infiltration models.

SI. No.	Models	(Mean)Range			Ranking Based on r/RMSE/NSE Values	AIC	
		r	RMSE (mm)	NSE		Range	Rank
1	Kostiakov-Lewis	(0.9988) 0.9897–1.0000	(**1.3710**) 0.1863–5.4202	(0.9975) 0.9791–0.9999	4	−5.27–136.81	4
2	Horton	(0.997) 0.9623–1.0000	(3.3654) 0.2117–45.9205	(0.9920) 0.9260–0.9999	6	2.64–237.04	6
3	Philip Two-Term	(0.9962) 0.9622–0.9999	(2.1918) 0.2825–7.2807	(0.9779) 0.7569–0.9999	5	10.71–149.21	5
4	Kutílek and Krejča	(0.9963) 0.9622–1.0000	(2.0352) 0.2534–7.2807	(0.9781) 0.7569–1.0000	**1**	8.25–149.21	**1**
5	Swartzendruber	(0.9988) 0.9900–0.9999	(1.5892) 0.1523–8.1250	(0.9975) 0.9801–0.9998	2	−14.16–153.82	2
6	Stroosnijder	(0.9929) 0.9622–0.9998	(4.1198) 0.2824–16.7037	(0.9744) 0.7569–0.9996	7	10.70–192.54	7
7	Brutsaert	(**0.9990**) 0.9955–0.9999	(1.4934) 0.1473–7.2807	(**0.9979**) 0.9910–0.9999	3	−15.62–149.21	**3**

**Note:** Boldfaced values indicate highest values of r and NSE and lowest value of RMSE; Ranks are based on the best performance of the models at majority grids.

According to the NSE values obtained, the Brutsaert model has the highest mean NSE value of 0.9970, ranging from 0.9910 to 0.9999 (Table 2). Similarly, it is also clear from Table 2 that efficacy of the Kostiakov-Lewis and the Swartzendruber models is excellent having NSE values ranging from 0.9801 to 0.9998 and from 0.99791 to 0.9999, respectively. On the other hand, the Philip Two-Term and Stroosnijder models perform relatively poor with NSE values ranging from 0.7569 to 0.9999 and from 0.7569 to 0.9996, respectively. Both of these models show lesser NSE values (≤0.8100) at two grids (Grids 3 and 9) falling in the north-western part of the study area. As far as the values of correlation coefficients (r) are concerned, all the models obtained similar ranks to those obtained with respect to model efficiency (NSE), though they show very high values of r (>0.96) at all the locations/grids. These infiltration models were ranked for each grid and the overall ranking of the infiltration models was decided according to their performances based on r, RMSE, and NSE at a majority of the grids. Accordingly, the performance of the infiltration models is ranked as Kutílek and Krejča > Swartzendruber > Brutsaert > Kostiakov-Lewis > Philip Two-Term > Stroosnijder model based on r/RMSE/NSE.

As to the AIC values, they vary from −5.27 to 136.81 for the Kostiakov-Lewis, from 2.64 to 237.04 for the Horton, from 10.71 to 149.21 for the Philip Two-Term, from −14.16 to 153.82 for the Swartzendruber, from 10.70 to 192.54 for the Stroosnijder and −15.62 to 149.21 for the Brutsaert models at all the locations (Table 2). In this study, the model having the least AIC value is considered to be the best model. As mentioned above, all the infiltration models were ranked grid-wise, and based on their performance at a majority of the grids, they were ranked for the whole study area. Thus, the Kutílek and Krejča model ranks 1^st^ having the least AIC values at a majority of the grids (about 30% of the sites) as compared to other models having AIC values in the range of 8.25 to 149.21. On the other hand, the Stroosnijder model performs the worst among all the models with the largest AIC values at most of the sites (>50% of the grids). Finally, performance of the infiltration models based on the AIC values is ranked as Kutílek and Krejča > Swartzendruber > Brutsaert > Kostiakov-Lewis > Philip Two-Term > Horton > Stroosnijder.

From the above results, it is revealed that the three-parametric process-based models (i.e., the Kutílek and Krejča, Brutsaert and Swartzendruber) rank higher in reproducing the cumulative infiltration at all the locations. The best performance of the Swartzendruber and Brutsaert models dovetails with the findings of Shukla *et al*.^26^, Nie *et al*.^19^ and Jačka *et al*.^17^ for different land management practices and soil types. Similarly, superior performance of these two models is consistent with an earlier study conducted in agricultural fields located in the lateritic terrain^22^.

### Estimates of saturated hydraulic conductivity

#### Statistical distribution

A statistical summary of the model-estimated saturated hydraulic conductivity (K_sat_) values as well as field-measured *K*_*fs*_ at all the sites is presented in Table 3. These measured and estimated saturated hydraulic conductivity values were subjected to the Kolmogorov-Smirnov test for finding out their best-fit distribution based on the lowest value of the Kolmogorov-Smirnov statistics. The best-fit distribution for measured and estimated K_sat_ along with their corresponding values of Kolmogorov-Smirnov statistics at 5% level of significance are shown in Table 3. According to this test, all the parameters including *K*_*fs*_ could be best described by a log-normal distribution except those estimated by the Philip Two-Term, Kutílek and Krejča, and Stroosnijder models (i.e., *A, K*_*sk*_ and *K*_*str*_). These three parameters can be best described by normal distributions.

**Table 3:** Spatial variability of saturated hydraulic conductivity estimated by seven infiltration models, field measurement and laboratory measurement.

SI. No.	Infiltration Models	Parameters	Basic Statistics			Best Distribution	
			Range	Mean ± SD	CV (%)	K-S Statistics	Distribution
1	Kostiakov-Lewis	*i*_*b*_ (mm/min)	0.00–1.28	0.19 ± 0.33	**173.7**	0.27	Log-normal
2	Horton	*i*_*c*_ (mm/min)	0.00–1.97	0.36 ± 0.48	134.7	0.12	Log-normal
2	Philip Two-Term	*A* (mm/min)	0.00–1.79	0.30 ± 0.46	153.3	0.26	Normal
3	Brutsaert	*K*_*b*_ (mm/min)	0.00–1.79	0.33 ± 0.47	142.4	0.10	Log-normal
4	Swartzendruber	*K*_*sw*_ (mm/min)	0.00–1.83	0.35 ± 0.48	137.1	0.10	Log-normal
5	Kutílek and Krejča	*K*_*sk*_ (mm/min)	0.00–1.92	0.37 ± 0.52	140.5	0.24	Normal
6	Stroonsnijder	*K*_*str*_ (mm/min)	0.00–1.92	**0.41** ± 0.53	129.3	0.26	Normal
7	Field Measurement	*K*_*fs*_ (mm/min)	0.01–1.75	0.37 ± 0.47	127.0	0.11	Log-normal
8	Lab Measurement	Measured *K*_*s*_ (mm/min)	0.00–3.10	0.22 ± 0.57	256.0	0.09	Log-normal

**Note:** K-S statistics: Kolmogorov-Smirnov statistics; −ve and +ve bias values indicate under predictions and over predictions, respectively; Boldfaced values indicate highest values of the statistics.

#### Model performance in predicting K_sat_

All the model-estimated K_sat_ values demonstrate high spatial variability over the lateritic terrain as indicated by very high values of coefficient of variation (CV > 129%). Among all the infiltration models, the Kostiakov-Lewis model yields the lowest value of mean K_sat_ (i.e., 0.19 ± 0.33 mm/min) with the highest CV of 173.7%, while the Stroosnijder model yields the highest K_sat_ value (*K*_*str*_ = 0.41 ± 0.53 mm/min) with the lowest CV of 129.3%. Further, the ANOVA test conducted over the log-transformed K_sat_ estimates (viz., *i*_*b*_, *i*_*c*_, *A*, *K*_*b*_, *K*_*sw*_, *K*_*sk*_, and *K*_*str*_) revealed that the differences in the mean values of all these parameters are strongly significant at 1% level of significance. Diverse assumptions of these models and field conditions contribute to these discrepancies in model predictions. Similarly, the estimated mean K_sat_ values yielded by a given model also demonstrate a high spatial variability at 1% level of significance. The Tukey HSD test further revealed that the mean value of *i*_*b*_ differs significantly from the mean values of *i*_*c*_, *K*_*b*_, and *K*_*sw*_ at 5% level of significance, while *A* varies significantly from the K_sat_ values estimated by other models except those estimated by the Kostiakov-Lewis (i.e., *i*_*b*_) and Kutílek and Krejča (i.e., *K*_*sk*_) models at 1% level of significance. When compared with *K*_*fs*_ and ‘measured *K*_*s*_’, difference in the measured and the model-estimated K_sat_ values is found to be not significant within 95% confidence limit, except *i*_*b*_ and *A*.

**Figure 9:**
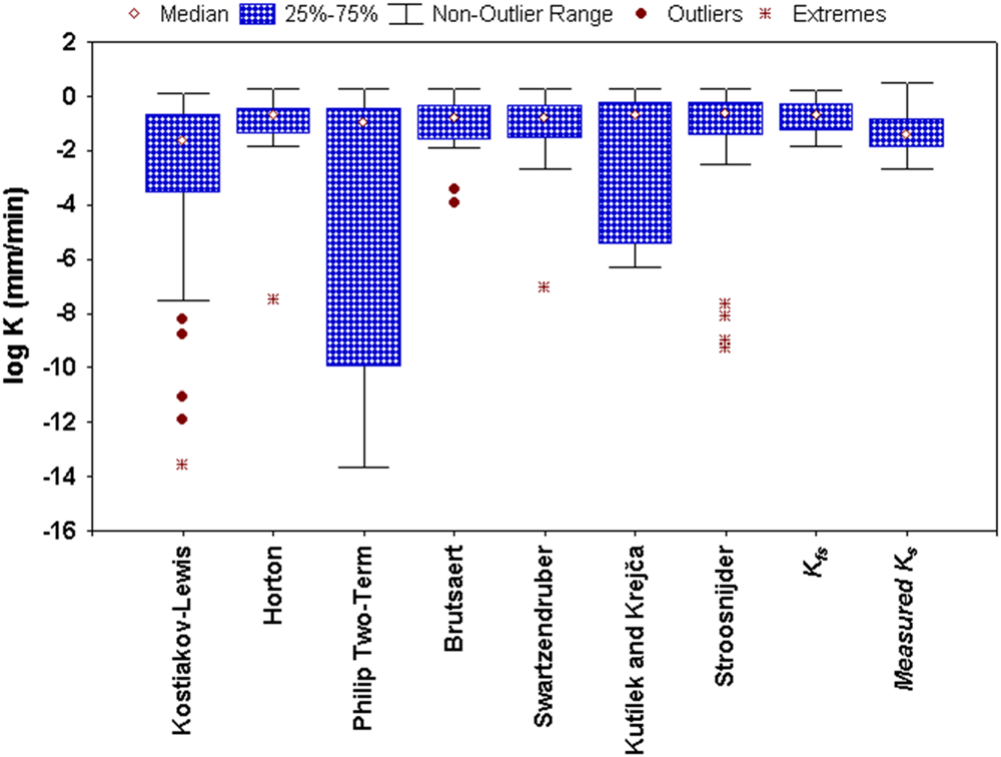
Box plot of the log-transformed saturated hydraulic conductivity values estimated by the infiltration models and the measured values (i.e., ‘*K*_*fs*_’ and ‘measured *K*_*s*_’).

All the seven infiltration models yield more or less under predicted or over predicted values of K_sat_ as compared to the measured values (i.e., ‘*K*_*fs*_’ and ‘measured *K*_*s*_’), except Kutílek and Krejča model when compared to ‘*K*_*fs*_’ (Table 3). At most of the grids/locations (>50%), however, the models yield over predicted K_sat_ against ‘*K*_*fs*_’, while mostly underpredict as opposed to the ‘measured *K*_*s*_’ values. The mean values of estimated K_sat_ are 0.19 mm/h for the Kostiakov-Lewis model, 0.36 mm/min for the Horton model, 0.30 mm/min for the Philip Two-Term model, 0.33 mm/min for the Brutsaert model, 0.35 mm/min for the Swartzendruber model, 0.37 mm/min for the Kutílek and Krejča model, and 0.41 mm/min for the Stroosnijder model as compared to the mean observed values of 0.37 mm/min (*K*_*fs*_) and 0.22 mm/min (‘measured *K*_*s*_’) for all the locations. Further, the degree of dispersion and skewness of the model-estimated K_sat_ values as well as the measured K_sat_ values are illustrated in Fig. 9. It is apparent that the K_sat_ yielded by all the infiltration models exhibit large variations (highest in *A* and lowest in *K*_*b*_), mostly in the first quartile (0–25%) as depicted from the longer bottom whiskers. The position of the median values and the length of whiskers show the non-symmetric nature of the estimated K_sat_ and ‘measured *K*_*s*_’ datasets except ‘*K*_*fs*_’. The Kostiakov-Lewis model estimated *i*_*b*_ shows the greatest deviation from the median *K*_*fs*_ with the lowest value. Overall, all the infiltration models, except the Kostiakov-Lewis and Philip Two-Term models, yield closer mean and median K_sat_ values when compared with *K*_*fs*_, as apparent from Table 3 and Fig. 9. This finding is also supported by the results of Tukey HSD test.

Moreover, the results of statistical evaluation (MAE, RMSE and NSE) of the models for predicting K_sat_ (when compared with *K*_*fs*_) revealed excellent performance of all the infiltration models with NSE ≥ 0.90 in predicting K_sat_, except the Kostiakov-Lewis model (Table 4). Among all the models, the Swartzendruber model performs the best, having the highest model efficiency (NSE = 0.985) and the least error values (MAE = 0.040 mm/min and RMSE = 0.057 mm/min). The Brutsaert model also estimates K_sat_ values with better values of the statistical indicators (NSE = 0.982; MAE = 0.046 mm/min and RMSE = 0.063 mm/min) which are close to those of the Swartzendruber model. On the other hand, the Kostiakov-Lewis model performs the worst in predicting K_sat_ having the lowest NSE of 0.386 and the highest errors, MAE and RMSE values of 0.179 mm/min and 0.364 mm/min, respectively. These results are consistent with those suggested by some of the past studies^17,22,25^ conducted in different land management practices and topographic settings, thereby indicating the robustness of the process-based, three-parametric models like the Brutsaert and the Swartzendruber models.

**Table 4:** Goodness-of-fit statistics of the infiltration models for predicting saturated hydraulic conductivity.

SI. No.	Models	MAE (mm/min)	RMSE (mm/min)	NSE
1	Kostiakov-Lewis	0.179	0.364	0.386
2	Horton	0.066	0.167	0.871
3	Philip Two-Term	0.080	0.105	0.949
4	Kutílek and Krejča	0.080	0.116	0.938
5	Swartzendruber	**0.040**	**0.057**	**0.985**
6	Stroosnijder	0.055	0.084	0.967
7	Brutsaert	0.046	0.063	0.982

As to the influence of texture and land use/land cover (LULC) on the model-estimated K_sat_ values, there exists a significant difference at 5% level of significance among the means of the parameters for a particular soil and LULC class except the lands having shrubland and vegetation. However, the difference in the mean values of K_sat_ estimated by a specific infiltration model is insignificant at 5% level of significance for different soil types and LULC classes.

#### Results of uncertainty analysis

**Figure 10:**
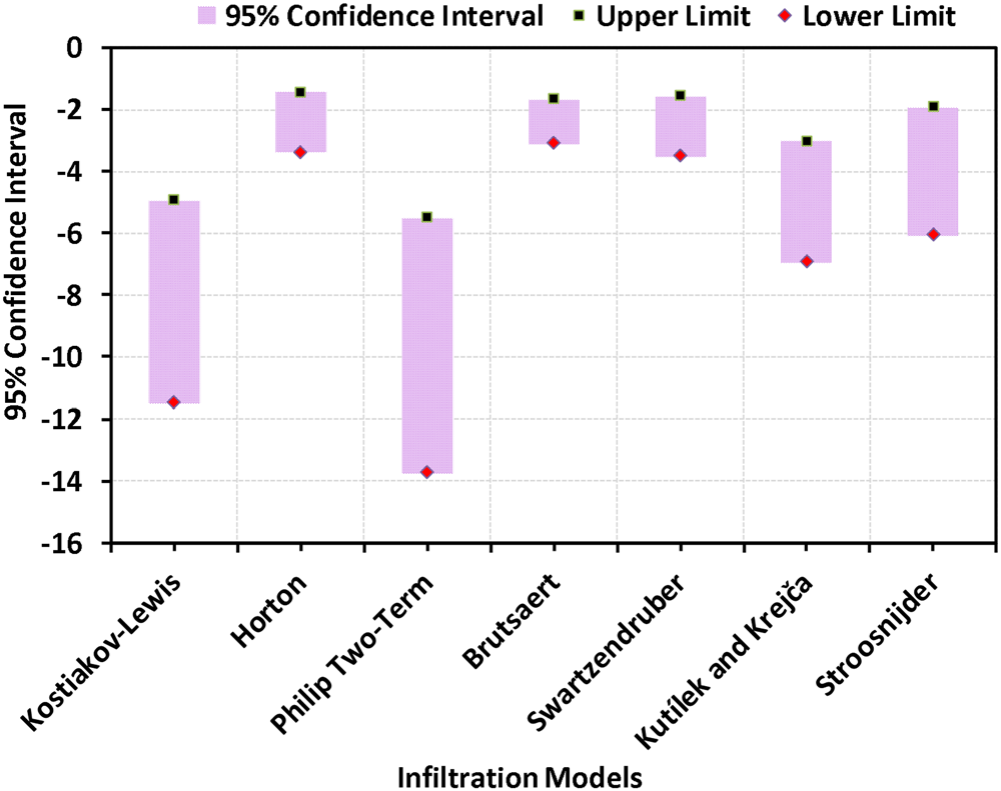
95% confidence intervals of the model-estimated saturated hydraulic conductivity (log-transformed) values along with their upper and lower limits for the study area.

**Figure 11:**
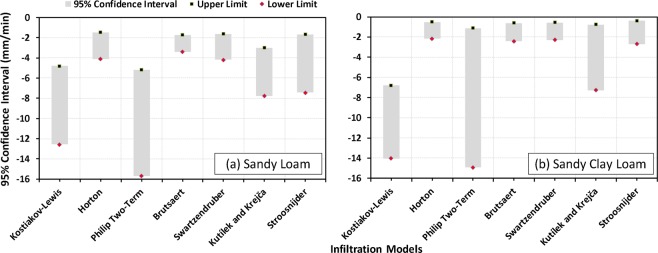
95% confidence intervals of the model-estimated saturated hydraulic conductivity (log-transformed) values along with their upper and lower limits (indicated as square and diamond shaped markers) for two soil textures (**a**) Sandy Loam, and (**b**) Sandy Clay Loam.

**Figure 12:**
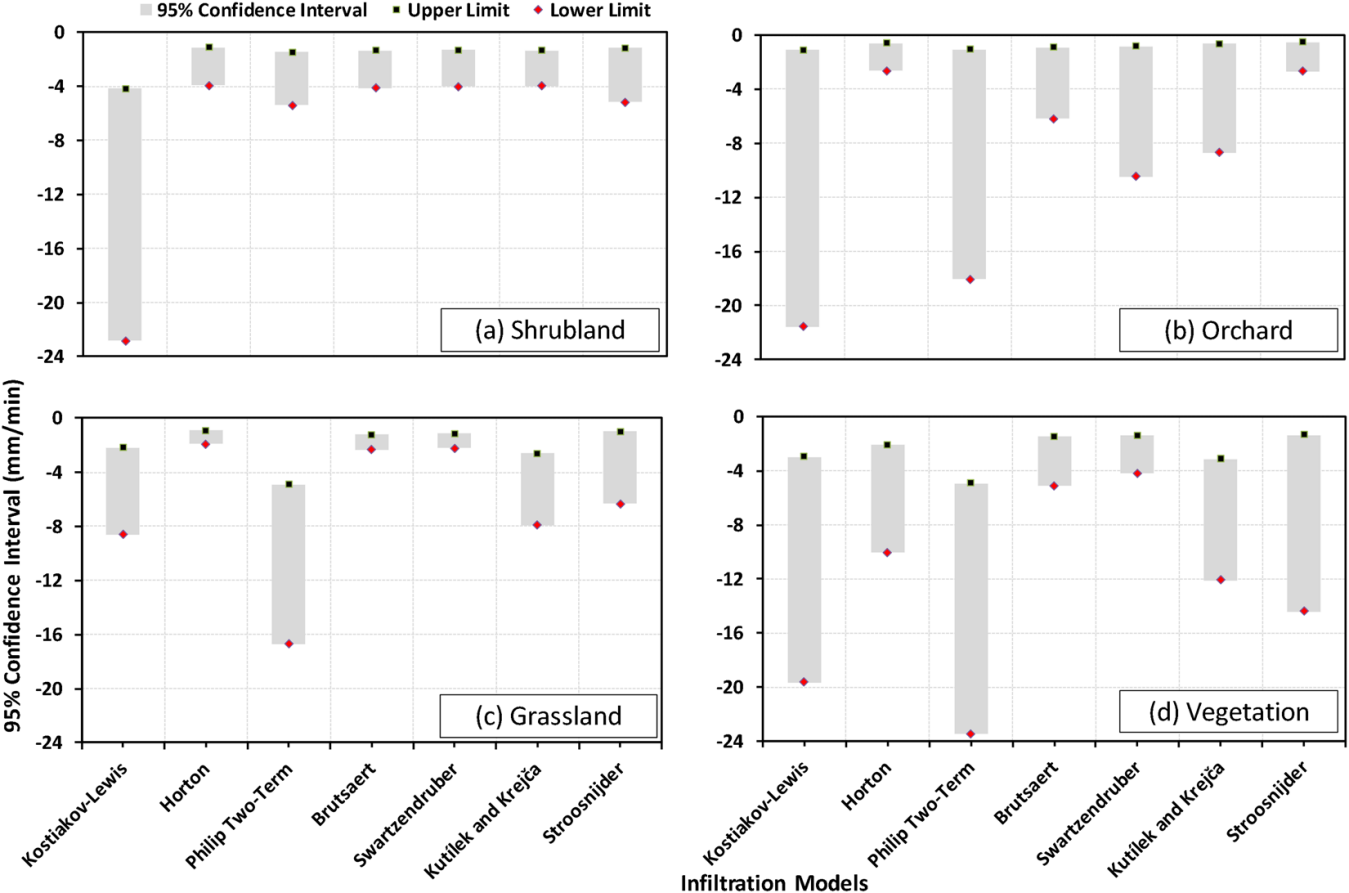
95% confidence intervals of the model-estimated saturated hydraulic conductivity (log-transformed) values along with their upper and lower limits (indicated as square and diamond shaped markers) for four land use/land cover classes (**a**) Shrubland, (**b**) Orchard, (**c**) Grassland, and (**d**) Vegetation.

The uncertainty analysis is aimed at determining the uncertainty associated with each infiltration model in estimating saturated hydraulic conductivity (K_sat_). The results of the bootstrap uncertainty analysis for the entire study area are shown in Fig. 10. The 95% confidence intervals of the model-estimated K_sat_ values with respect to different soil textures and land use/land cover are shown in Figs. 11(a,b) and 12(a–d), respectively. It is obvious from Fig. 10 that the Horton, Brutsaert and Swartzendruber models have narrower 95% probability bands for the estimated K_sat_ (log-transformed) values compared to the rest of the infiltration models. In particular, the Brutsaert model estimates K_sat_ with the least uncertainty as revealed from the least distance between the upper (−1.68 mm/min) and lower (−3.11 mm/min) limits of the 95% confidence intervals around the mean of log-transformed K_sat_ values (Fig. 10). The Swartzendruber and Horton models have similar level of uncertainty with 95% confidence interval bandwidths of 1.93 and 1.96 mm/min, respectively. In contrast, the Phillip Two-Term model has the highest uncertainty (lower limit: −13.7 mm/min and upper limit: −5.51 mm/min) followed by the Kostiakov-Lewis model (lower limit: −11.5 mm/min and upper limit: −4.95 mm/min), Stroosnijder model (lower limit: −6.07 mm/min and upper limit: −1.93 mm/min) and the Kutílek and Krejča (lower limit: −6.95 mm/min and upper limit: −3.04 mm/min) model in estimating K_sat_ values. The least uncertainties associated with the Brutsaert and Swartzendruber models might be due to the fact that they are improved versions of Philip’s infinite series solution and three-parametric process-based equations. Therefore, they have greater fitting abilities for almost all the sites over the study area as compared to other two-parametric models. However, the sensitivity of the Kutílek and Krejča model to soil heterogeneity results in higher uncertainties in the K_sat_ values, in spite of being a process-based model and having three parameters.

As to the uncertainty of the model-estimated K_sat_ for different soil types, the Horton, Brutsaert and Swartzendruber models show the least uncertainty in predicting K_sat_ compared to other infiltration models for both types of soils (Fig. 11(a,b)). The widths of 95% confidence bands of K_sat_ estimated by these three models are about 2.64, 1.69, 2.57 mm/min for sandy loam and 1.65, 1.83, 1.75 mm/min for sandy clay loam soils, respectively. It is also evident that the 95% confidence bands yielded by the Kostiakov-Lewis, Horton, Swartzendruber and Stroosnijder models are wider for sandy loam type soil (Fig. 11(a)) than the sandy clay loam type soil (Fig. 11(b)). The Brutsaert model yields K_sat_ with almost the same uncertainty for both the soils having 95% confidence bandwidth of about 2 mm/min (Fig. 11(a,b)). The Stroosnijder model, however, estimates K_sat_ for the sandy clay loam soil with very less uncertainty (Fig. 11(b)) as compared to its performance in the study area as a whole (Fig. 10).

On the other hand, the uncertainty of the infiltration models in predicting K_sat_ for different types of land use/land cover (LULC) is discernible from Fig. 12(a–d), which reveals that the Brutsaert, Swartzendruber and Horton models yield K_sat_ values more precisely and consistently for all types of land use/land cover, except the orchard type LULC. Specifically, the Brutsaert (−2.37 mm/min, −1.23 mm/min), Swartzendruber (−2.22 mm/min, −1.16 mm/min) and Horton (−1.95 mm/min, −0.92 mm/min) models have the highest accuracy for grassland type LULC in estimating K_sat_ (Fig. 12(c)). However, it is also apparent that each infiltration model, except Brutsaert is sensitive to a particular LULC in estimating K_sat_. For instance, the Swartzendruber model predicts K_sat_ with a higher uncertainty (lower limit of −10.5 mm/min and an upper limit of −0.84 mm/min) for the orchard type LULC (Fig. 12(b)). The Philip Two-term and Kostiakov-Lewis models estimate K_sat_ with the least uncertainty for shrubland (95% confidence bandwidth: 3.96 mm/min) and grassland (95% confidence bandwidth: 6.41 mm/min), respectively as compared to other LULC types. Similarly, the uncertainty associated with the Kutílek and Krejča model is smaller for the shrubland (lower limit: −3.96 mm/min, upper limit: −1.35 mm/min), while the uncertainty associated with the Stroosnijder model is the highest for the vegetation type LULC (lower limit: −2.67 mm/min, upper limit: −0.51 mm/min) compared to their performance for other types of LULC.

## Conclusions

In the present study, 33 grid-based (400 m × 400 m) double ring infiltration experiments were carried out in a tropical sub-humid region of Eastern India. The grid-specific infiltration data sets obtained were used to investigate infiltration characteristics and evaluate the efficacy of seven infiltration models in predicting cumulative infiltration. Additionally, grid-wise soil physical properties and saturated hydraulic conductivity (K_sat_) of the vadose-zone were determined in the laboratory along with detailed land use/land cover (LULC) mapping. The capability of the infiltration models was also evaluated in predicting infiltration behavior and estimating K_sat_. Finally, a non-parametric Bootstrap uncertainty analysis was carried out to quantify uncertainties associated with K_sat_ estimates given by the infiltration models for the entire study area as well as for different LULC and soil types. The major findings of this study are as follows:

- The results of the detailed field investigation indicated that the average ‘quasi-steady infiltration rate’ of the study area is 22.23 ± 28.27 mm/h, which varies appreciably (CV = 127%) over the area. About 96% of the study area falls under ‘*low*’ and ‘*medium*’ infiltration rate categories.
- Infiltration characteristics of the study area is not directly dependent on soil texture, bulk density and land use/land cover patterns only. It is also influenced by other factors such as presence of macropores and complex flow dynamics in vadose zones, which are typical features of the lateritic vadose zones of tropical sub-humid regions. Further investigations in this direction is recommended under different agro-climatic settings.
- All the seven infiltration models are capable of predicting cumulative infiltration at all the sites with moderate to excellent model performance (NSE = 0.76 to 1.00 and RMSE = 0.15 to 16.70 mm). However, the cumulative infiltration time series predicted by the Stroosnijder and Philip Two-Term models deviate from the measured cumulative infiltration time series at some sites. Overall, the performance of the infiltration models in predicting cumulative infiltration of the study area is ranked as Kutílek and Krejča > Swartzendruber > Brutsaert > Kostiakov-Lewis > Philip Two-Term > Horton > Stroosnijder.
- K_sat_ estimates yielded by the seven infiltration models exhibit a significant spatial variation. Comparison of K_sat_ estimates with the field measured *K*_*fs*_ values indicated that except the Kostiakov-Lewis model, all the infiltration models yield reliable estimates of K_sat_ with the Brutsaert and the Swartzendruber models yielding K_sat_ values closest to *K*_*fs*_ measured in the lateritic terrain under study.
- The uncertainty analysis of the infiltration models applied to the entire area revealed that the Brutsaert model estimates K_sat_ most accurately with the least width of the 95% confidence band (1.43 mm/min), followed by the Swartzendruber (1.93 mm/min) and Horton (1.96 mm/min) models. In contrast, the Phillip Two-Term model has the widest 95% confidence band (8.23 mm/min) followed by the Kostiakov-Lewis, Stroosnijder and Kutílek and Krejča models, thereby indicating their poor prediction capabilities.
- Uncertainty analysis of the models for different soil types and LULC indicated that the Horton, Brutsaert and Swartzendruber models predict K_sat_ with the least uncertainty for all soil types. On the other hand, the Kostikov-Lewis, Horton, Philip Two-Term, Swartzendruber, Kutílek and Krejča, and Stroosnijder models are sensitive to grassland, vegetation, shrubland, orchard, shrubland, and vegetation type grids, respectively. The Brutsaert model, however, estimates K_sat_ with similar uncertainties irrespective of soil types and LULC.

Detailed information about the spatial variability of infiltration characteristics, quantification of saturated hydraulic conductivity and soil physical properties as well as the results of infiltration modeling obtained through this study are helpful in better understanding of complex hydrological processes and their spatial variability in vadose-zone systems of tropical sub-humid/humid regions. The results obtained in this study can provide a scientific basis for the efficient planning and design of irrigation and surface drainage systems to address the recurrent flood and drought problems in the region, including the study area. In addition, these findings are also useful for the proper planning and design of rainwater harvesting and artificial recharge structures to address growing water scarcity and environmental problems in the region under changing climatic and socio-economic conditions. The methodology presented in this study including uncertainty analysis can facilitate a precise selection of infiltration models for the reliable prediction of saturated hydraulic conductivity under different land use/land cover and soil types of the region under study as well as in other regions. It is recommended that such a study be carried out at a catchment scale for better irrigation-water management and accurate assessment of water balance components, which in turn can help planners and water managers in the  sustainable management of water and land resources.

## Data availability

The data may be available on request to the corresponding author.
